# The relationship between self-reported physical frailty and sensor-based physical activity measures in older adults – a multicentric cross-sectional study

**DOI:** 10.1186/s12877-022-03711-2

**Published:** 2023-01-24

**Authors:** Stephanie Schmidle, Philipp Gulde, Raphael Koster, Cristina Soaz, Joachim Hermsdörfer

**Affiliations:** 1 Human Movement Science, Department of Sport and Health Sciences, Technical University of Munich, Munich, Germany; 2 MADoPA, Centre Expert en Technologies et Service pour le Maintien en Autonomie á Domicile des Personnes Agées, Paris, France; 3 Qolware GmbH, Munich, Germany

**Keywords:** Frailty, Ageing, Assessment, Self-report, Accelerometry, Actigraphy, Physical activity

## Abstract

**Background:**

The decline in everyday life physical activity reflects and contributes to the frailty syndrome. While especially self-reported frailty assessments have the advantage of reaching large groups at low costs, little is known about the relationship between the self-report and objective measured daily physical activity behavior. The main objective was to evaluate whether and to what extent a self-reported assessment of frailty is associated with daily physical activity patterns.

**Methods:**

Daily activity data were obtained from 88 elderly participants (mean 80.6 ± 9.1 years) over up to 21 days. Acceleration data were collected via smartwatch. According to the results of a self-report frailty questionnaire, participants were retrospectively split up into three groups, F (frail, *n* = 43), P (pre-frail, *n* = 33), and R (robust, *n* = 12). Gait- and activity-related measures were derived from the built-in step detector and acceleration sensor and comprised, i.a., standard deviation of 5-s-mean amplitude deviation (MADstd), median MAD (MADmedian), and the 95th percentile of cadence (STEP95). Parameters were fed into a PCA and component scores were used to derive behavioral clusters.

**Results:**

The PCA suggested two components, one describing gait and one upper limb activity. Mainly gait related parameters showed meaningful associations with the self-reported frailty score (STEP95: R^2^ = 0.25), while measures of upper limb activity had lower coefficients (MADmedian: R^2^ = 0.07). Cluster analysis revealed two clusters with low and relatively high activity in both dimensions (cluster 2 and 3). Interestingly, a third cluster (cluster 1) was characterized by high activity and low extent of ambulation. Comparisons between the clusters showed significant differences between activity, gait, age, sex, number of chronic diseases, health status, and walking aid. Particularly, cluster 1 contained a higher number of female participants, whose self-reports tended towards a low health status, the frequent use of a walking aid, and a higher score related to frailty questions.

**Conclusions:**

The results demonstrate that subjective frailty assessments may be a simple first screening approach. However, especially older women using walking aids may classify themselves as frail despite still being active. Therefore, the results of self-reports may be particularly biased in older women.

## Introduction

Frailty is commonly described as a condition, which increases the risk for adverse health outcomes (such as sarcopenia, fractures, and death) even after exposure to minor stressors [1, 2]. Within this context, frailty is seen as a transitional state in the dynamic progression of functional decline [3]. Until now, the causes of frailty are not fully understood [4]. While frailty is considered to be age-associated, it is not necessarily age-dependent and often, but not exclusively, occurs as a comorbidity of specific diseases. However, frailty can also be present in the absence of disease and not all affected elderlies experience the same symptoms [4, 5]. The vulnerability of the population and its multifaceted burden is high. Therefore, regular screenings are recommended [1]. However, limited resources (e.g., lack of time) constitute barriers to a frequent and comprehensive frailty-screening in primary care settings [6, 7]. In addition, frailty is an entity that clinicians derive from a variety of symptoms, which impedes diagnosis [3]. As a result, a definitive and consent operational definition remains still unspecified [6].

There is a plethora of frailty assessments [8], which can be divided into two main subcategories: a) the frailty phenotype instruments, which focus primarily on measuring motor function as well as activity and result in a categorical score from robust to frail (e.g., frailty phenotype [9]), or b) frailty index instruments that add a variety of factors (up to 70), resulting in a continuous scale with higher frailty scores for a greater number of ‘conditions’ [8, 10] (e.g., deficit accumulation approach – Frailty Index [11]). Further, these assessments can be categorized as subjective (only self-reported items), objective, and mixed [12]. While objective performance-based instruments offer several advantages, such as more precise and valid results and an increased sensitivity to changes over time, scoring methods based on observation and subjective assessments are typically quick and easy to administer. They can assess complex behavior but lack objectivity. A special case of self-report (subjective) assessments are questionnaires, which can be an effective way to reach larger groups at low costs [12, 13]. These questionnaires are well accepted, impose a relatively low burden on the individual, and do not interfere much with usual habits [13]. However, those self-reports are generally prone to a diversity of biases, e.g., perceptual errors and memory biases [14]. Nevertheless, self-reports might be especially useful for initial screening purposes [15]. In general, assessments should be brief and simple in order to increase the chance of implementation into clinical practice and, thus, should be chosen by indication: screening for the presence or level of frailty, tracking the dynamic changes in frailty, or with the goal of monitoring therapeutic interventions [1].

An older individual’s level of physical activity (PA) constitutes an important criterion of frailty [16]. While a decline in everyday life PA may both reflect and contribute to frailty, regular PA is thought to play an important role as a preventive factor and, thus, could positively influence the continuum [17]. Besides self-reports and simple clinical tests (e.g., strength or gait speed), a variety of tools already exists to assess PA at different levels of frailty, including accelerometers, heart rate monitors (HR), portable electromyography devices (EMG), and global positioning systems (GPS) [18]. In particular, portable sensors, also called wearables, offer the possibility to measure activity in everyday life in a simple, inexpensive, unobtrusive, and reliable way [19]. There is a wide range of evidence showing that activity levels detected by sensors (e.g., sedentary behavior, light and moderate-to-vigorous physical activity levels) are strongly associated with the frailty status [20] and can discriminate between different levels of frailty (e.g., [18, 19, 21–23]). In particular, the wearable-derived number of steps and duration of activity measured with accelerometers seem to be more strongly correlated with the level of frailty than other measures (e.g., number of bursts in EMG and gait speed assessed via GPS) [18]. In 2021, Wanigatunga and colleagues [24] investigated the associations between accelerometer-derived patterns of routine daily physical activity and phenotypic frailty. They showed that unfavorable activity patterns (fewer active minutes, more sedentary minutes, lower activity counts, and higher activity fragmentation) predicted an increased likelihood of frailty [24]. In fact, there has been a marked increase in the amount of information on the association between daily activity and physical frailty as measured by mostly mixed assessments. Different from these approaches, self-reported frailty assessments have the advantage of reaching larger groups and achieving high response rates, and thus can be used as a simple first step screening tool [15, 25]. Nunes and colleagues [15], for instance, compared the phenotype frailty score with a self-reported instrument and concluded that the latter can be used as simple, rapid, and low-cost screening tool offering a satisfactory level of reliability and sensitivity [15]. To date, little is known about the relationship between an individual’s exclusively self-reported frailty status and objective measures of daily physical activity behavior. Since most of the questionnaire items are including PA aspects, one might use PA to investigate the accordance with the actual movement behavior. Therefore, we aimed towards investigating the relationship between different aspects of sensor-based daily activity measures, such as those mainly expressing gait activity (ambulation) and those mainly expressing upper limb activity, with the self-estimation of frailty in a cohort of elderly individuals potentially vulnerable to frailty. To better understand how objective measures of daily activity relates to self-reported frailty, we further analyzed behavioral patterns by deriving clusters to validate the self-reports. We decided to examine this relationship by using smartwatches, as wrist-worn assessments have the advantage to capture more commonly performed tasks of daily living (e.g., cooking and housework) in addition to ambulation and might therefore provide a more comprehensive picture of total daily activity [26]. Based on previous reports, we hypothesized that objective gait function would be a stronger predictor of self-assessed frailty status than other sensor-based measures. Additionally, we hypothesized that the cluster analyses would reveal clusters of participants with meaningful differences concerning their behavioral patterns and health states.

## Materials and methods

### Sample and procedure

We recruited a convenience sample of 114 older adults within the EIT Health project ‘FRAIL – Frailty Assessment in Daily Living’. The FRAIL project aimed towards improving the supervision of frail elderly (e.g., health state and falls). The recruitment took place in Germany and France between May and November 2019. Within the project, older adults living in nursing homes, assistive living environments, and private homes were approached to participate. Within this project, two key persons were in charge for the recruitment process; one in Germany and one in France. In both countries, the recruitment of the older adults within the nursing homes and assistive living environments was done by the respective care manager after being contacted and informed about the inclusion and exclusion criteria by the recruitment organizers. In France, a total of four nursing homes were recruited, whilst in Germany two nursing homes were involved (with one care manager per institution, *n* = 6). The contact to older people living in private homes was done by dissemination actions in public forums. For this purpose, the study was presented during senior citizen days and older adults had the opportunity to sign up in a contact list. Later, those individuals were contacted by their respective recruiting organizers. The inclusion criteria were a minimum age of 60 years and the basic understanding of the operations relevant to the measures of the watch. Exclusion criteria were pronounced parchment skin with increased risk of injury due to the smartwatch, diseases with cognitive impairment, or dementia which prevented understanding of the informed consent and usage of the smartwatch. An important issue was the ability to handle the smartwatch with two major challenges: continuous data collection and regular recharging. Unfortunately, not all buttons of the smartwatch could be deactivated which increased the risk of an unintentional termination of the recording. Furthermore, the smartwatch had to be charged once to twice a day. We collected a questionnaire covering the categories ‘health status’, ‘personal environment’ (e.g., sociodemographic and physical condition), and ‘frailty status’ and equipped all participants with a smartwatch for up to 21 days. The measurement procedure took place in the respective living environment of the participants:

In a first step, the examiner explained the technical device (smartwatch) and participants signed the informed consent. In the next step, the participants were asked to complete the questionnaire together with the trained examiner (i.e., the respective recruitment organizer). To increase compliance, participants were given the choice of wrist. This was based on the findings of Dieu and colleagues [27], who showed that counts do not profoundly differ between the dominant and the non-dominant wrist and both locations are well associated with counts derived from a sensor worn around the waist (both rs = 0.88) [27]. Therefore, 73 chose the left and 15 persons the right wrist. We asked participants to wear the smartwatch for up to 21 days. The start of the measurement was variable between subjects in terms of both day of the week and time of the day.

Participants were only included in the analysis if data was available for at least 6 measurement days of wear time within the 21 days period with a minimum of eight measurement hours between 08:00 am and 08:00 pm. With this inclusion criteria, we aimed to preserve reliability [28] and include as many patients as possible. This is based on the statement of Aadland & Ylvisåker [28] that a one-week measurement already shows acceptable-to-good reliability. Ethical approval was given by the ethics committee of the Medical Faculty of Technical University of Munich. All participants provided written informed consent. A post hoc power analysis, using the weakest derived OR (2.93), resulted in a power of 0.96. This indicates a sufficient sample size.

### Measures

The measurements consisted of two components: the subjective frailty questionnaire, which included sociodemographic information and physical condition, and the continuous activity measurement with the smartwatch.

#### Sociodemographic and physical condition

Information on sociodemographic status and the physical condition was obtained from the following questions: (i) sex (male/female); (ii) age (in years); (iii) BMI (kg/m^2^); (iv) multimorbidity (number of chronic diseases), e.g., stroke, hypertonia, asthma, and multiple sclerosis; (v) health status (self-classified state of health): 1: excellent; 2: very good; 3: good; 4: fair; 5: poor; (vi) course of health status (self-rated health compared to one year ago): 1: much better now; 2: somewhat better now; 3: about the same; 4: somewhat worse now; 5: much worse now; (vii) feeling of safety at home: 1: fully; 2: quite a bit; 3: moderately; 4: slightly; 5: not at all; (viii) feeling of safety outside the home: 1: fully; 2: quite a bit; 3: moderately; 4: slightly; 5: not at all; (ix) living condition: living alone yes/no (if no, with whom); (x) use of walking aids: yes/no.

#### Subjective frailty assessment

The self-reported frailty status was assessed by a questionnaire [29] in accordance with Santos-Eggimann et al. [30]. The questionnaire was based on the five constructs from Fried’s frailty phenotype [9]: (1) unintentional weight loss: a) self-reported loss of appetite and b) decreased amount of food intake; (2) exhaustion: binary (yes/no) self-report response on whether the subject has too little energy to execute daily tasks; (3) low muscle strength: binary (yes/no) self-reported ability to lift or carry objects heavier than 5 kg; (4) low physical activity: self-reported frequency of engagement in moderate PA (e.g., gardening, cleaning the car or going for a walk); (5) weakness: self-reported flights of stairs that can be climbed without rest (a) one flight of stairs or b) several flights of stairs. The dimension coding for each criterion was rated as follows: (1) if the individual reported a loss of appetite or if the response was having eaten less than usual; (2) if the individual reported having lacked energy; (3) if the individual reported having difficulty carrying out the activity mentioned; (4) if the individual reported less than once a week; (5) if the individual reported limitations in one of the two activities proposed.

#### Sensor-based physical activity tracking

To collect objective physical activity data, we used a Huawei 2 (4G) smartwatch (Huawei watch 2 (4G), Huawei Technologies Co., Ltd., Shenzhen, China) at the participants’ wrist of choice. Data was sent regularly via mobile net to a main server using custom software. For step detection, the device’s built-in acceleration-based pedometer of the device was used. The accelerometer data was captured at a frequency of 100 Hz. For reporting different activities, a 5 second time period was used [31, 32] (i.e., 500 samples per 5 s period). Therefore, the vector magnitude (r) was calculated at each time point (i), followed by the mean vector magnitude for the 5 second time period ($\overline{r}$). This allowed the computation of the mean amplitude deviation (MAD) metric – which provides a measure of the intensity of acceleration changes, i.e., the intensity of PA, for every 5 seconds of data [33]:

$$Mean\ Amplitude\ Deviation\ (MAD)=\frac{1}{n}{\sum}_{i=1}^n\left|{r}_i-\overline{r}\right|$$

(where;)${r}_i=\sqrt{x^2+{y}^2+{z}^2}$ = *i*^*th*^ vector magnitude at each time point$\overline{r}$ = mean vector magnitude within the time period of interest*n* = number of data points of the time period.

The metric of MAD has been validated in multiple studies, e.g., Bazuelo-Ruiz [34] found strong associations of MAD with indirect calorimetry (r = 0.94). Additionally, the Huawei smartwatch has a reliable and sensitive acceleration sensor, which we were able to show in our previous study about kinematic analyses of ADL [35].

#### Data analysis

**Fig. 1:**
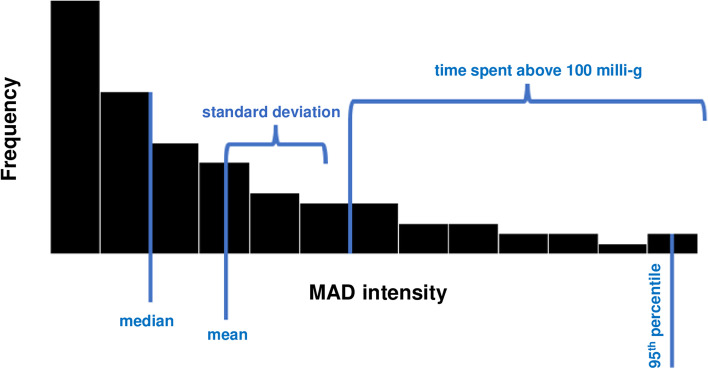
Graphical representation of the MAD-based activity parameters

The parameters derived from the smartwatch data comprised the mean MAD (MADmean), the standard deviation of MAD (MADstd), the relative MAD (MADrel), the median MAD (MADmedian), the 95% percentile of MAD (MAD95), the fragmentation rate of MAD (MADfrag), the 95% percentile of cadence (STEP95), and the average number of steps per 5 s (STEPmean). While we used the MAD-related parameters to assess the kinematic physical activity (see Fig. 1), the step-related parameters aimed to assess gait (ambulation). We used two frailty scores for analysis: the classical range from 0 to 5 including all 5 criteria of physical frailty (‘weight loss’, ‘exhaustion’, ‘muscle strength’, ‘PA’, and ‘weakness’) and a reduced version omitting the two parameters (‘muscle strength’ and ‘weight loss’) that cannot be assessed by a wrist worn sensor.

Mean MAD (MADmean): The mean of all MAD values in milli-g. Higher values indicate more physical acitivity. More intense physical activity is accounted for.

Standard deviation of MAD (MADstd): The variability of physical activity in milli-g. Higher values indicate a more variable physical activity (e.g., by phases of intense activity).

Relative MAD (MADrel): Relative amount time spent in MAD levels > 100 m-g. Higher values indicate more (health relevant) physical activity.

Median MAD (MADmedian): The median of all MAD values in milli-g. Higher values indicate more physical activity, independent from the height of the achieved maximum MAD levels. More intense physical activity is not accounted for.

95th percentile of MAD (MAD95): The 95. percentile of all MAD values in milli-g. Higher values indicate peaks of intense higher physical activity. Overall physical activity level is not accounted for.

Fragmentation rate of MAD (MADfrag): Standard deviation of the first derivative of the MAD time-series in milli-g/s. Higher values indicate higher fragmentation of physical activity. While this is commonly used to describe the relation of long and short bouts in ratio, this parameter targets the adaptation of energy expenditure, where higher values indicate task specific changes in intensities and are strongly associated with the total amount of physical activity.

95th percentile of cadence (STEP95): The 95. percentile of cadence in steps per minute. Higher values indicate a higher gait function. Overall gait related physical activity is not accounted for.

Average number of steps per 5 s (STEPmean): Average number of steps taken per 5 s. Higher values indicate more gait related physical acitvity.

### Statistical approach

In a first step, we assessed the relationship between the subjective questionnaire score and the acceleration-based parameters ‘STEP95’ and ‘MADmedian’ by means of Spearman correlation analyses. While the sensor-based parameters were highly intercorrelated (Fig. 4), the two parameters were used as a surrogate. Correlations were calculated for both frailty scores, the full score (0:5) and the reduced score (0:3). In a second step, we conducted a two-component confirmatory principal component analysis with a varimax rotation for the sensor-data (one component as gait/ambulation and one as upper limb activity) to calculate the participants’ component scores. Thresholds for the Kaiser-Meyer-Olkin sample adequacy was set to ≥0.50 and minimum communalities to ≥0.50. Based on the individuals’ component scores, a cluster analysis using k-medoid clustering was performed. The number of clusters was derived from a scree plot. Additionally, we generated a correlation matrix including all objective activity parameters, the subjective frailty scores, and the dimensions gait and activity. Third, cluster differences in terms of behavioral and person-related properties were tested using analyses of variance (one-way ANOVAs with Tukey post hoc tests) and, in case of feeling of security and health status, by means of Kruskal Wallis tests (Dunn’s pairwise comparison post hoc test). In case of sex, walking aid, and frailty status, we used the Pearson’s Chi-squared test (Fisher’s exact post hoc test with Bonferroni correction). Odds ratios and the 95% CI were calculated for sex and frailty distribution, as well as for each criterion separately. Furthermore, we calculated one-way ANOVAs comparing each subjective criterion with the objective activity data separately. Effect-sizes were expressed as eta squared η^2 ^Cramer’s V, Cliff’s Delta *d*, Cohen’s f^2^, and Cohen’s d. The threshold for critical variance inflation was set to 5.0, **α** was set to 0.05. All tests were run in R Studio (version 2021.09.2, RStudio Inc., Vienna, Austria).

## Results

**Fig. 2:**
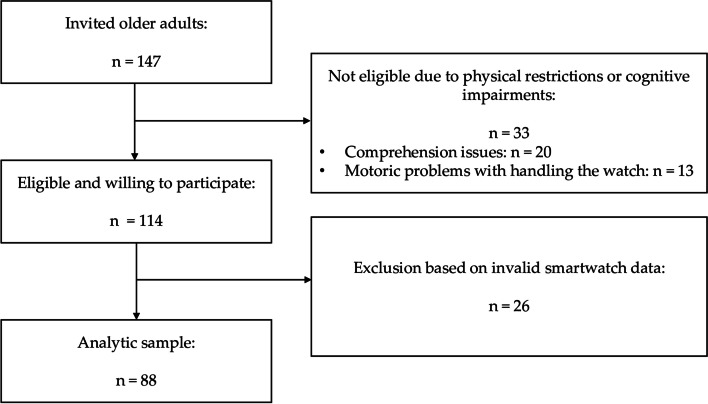
Flowchart of the recruitment procedure

In sum, a total of *n* = 88 participants (France: *n* = 37; Germany: *n* = 51) were included in the final analysis with a mean age of 80.6 years (range: 62 - 99 years) (Fig. 2). After an initial invitation of 147 older adults, 33 had to be excluded due to comprehension issues or motoric problems. 15 participants from Germany and 11 participants from France had to be excluded due to not meeting wear time-related inclusion criteria.

Of the total sample (*n* = 88), 13% people were robust, 38% were pre-frail, and 49% were frail. Weakness was the most prevalent frailty criterion (68%), whereas the weight loss criterion was the least prevalent (32%) one. In the French cohort, 18% of the participants were robust, 32% were pre-frail, and 50% were frail. In the German cohort, we observed a similar distribution, with 10% of participants being robust, 40% pre-frail participants, and 50% frail. A Pearson’s Chi-squared test comparing the distribution of frail, pre-frail, and robust participants between France and Germany did not show significant differences (*p* = 0.42) (Table 1).

**Table 1:** Demographics of the sample

Characteristic	Robust R, *n* = 12	Pre-frail P, *n* = 33	Frail F, *n* = 43	Total, *n* = 88
Female, n (%)	4 (33)	15 (45)	29 (67)	48 (55)
Age (years)	72.5 (5.8)	80.9 (9.3)	82.7 (8.6)	80.6 (9.1)
BMI (kg/m^2^)	25.6 (4.0)	25.8 (3.4)	27.4 (5.3)	26.5 (4.6)
Multimorbidity	0.9 (0.7)	1.2 (0.8)	1.5 (1.0)	1.3 (0.9)
Health status	2.6 (0.8)	3.1 (0.7)	3.9 (0.8)	3.4 (0.9)
Course health status	3.1 (0.8)	3.2 (0.7)	3.7 (1.1)	3.4 (0.9)
Feeling of safety (home)	1.3 (0.5)	1.5 (0.7)	1.7 (0.9)	1.6 (0.8)
Feeling of safety (outside)	1.6 (0.5)	2.0 (1.0)	2.3 (1.3)	2.4 (1.2)
Days of measurement	16.4 (5.2)	17.9 (4.8)	17.5 (5.4)	17.5 (5.1)
Living condition, n (%)				
Alone	2 (17)	7 (21)	12 (28)	21 (24)
Partner/relatives	7 (58)	13 (39)	16 (37)	36 (41)
Nursing home	3 (25)	13 (39)	15 (35)	31 (35)
Walking aid, n (%)	0 (0)	14 (42)	31 (72)	45 (51)
Frailty criteria, n (%)				
Unintentional weight loss	0 (0)	5 (15)	23 (53)	28 (32)
Exhaustion	0 (0)	6 (18)	32 (74)	38 (43)
Low muscle strength	0 (0)	13 (39)	35 (81)	48 (55)
Low physical activity	0 (0)	6 (18)	28 (65)	34 (39)
Weakness	0 (0)	20 (61)	40 (93)	60 (68)

Mean values, standard deviations. *Multimorbidity* mean number of comorbidities (0 - 9), *Health status* self-rated (best: 1; worst: 5), *Course health status* self-rated comparison to health 1 year ago (improvement: 1; deterioration: 5), *Feeling of safety* 1: fully; 5 not at all, *Unintentional weight loss* loss of appetite or decreased food intake, *Exhaustion* too little energy to execute daily tasks, *Low muscle strength* not able to carry objects of more than 5 kg, *Low physical activity* being engaged in at least moderate PA on less than once per week, *Weakness* not being able to take more than one flight of stairs without rest

**Fig. 3:**
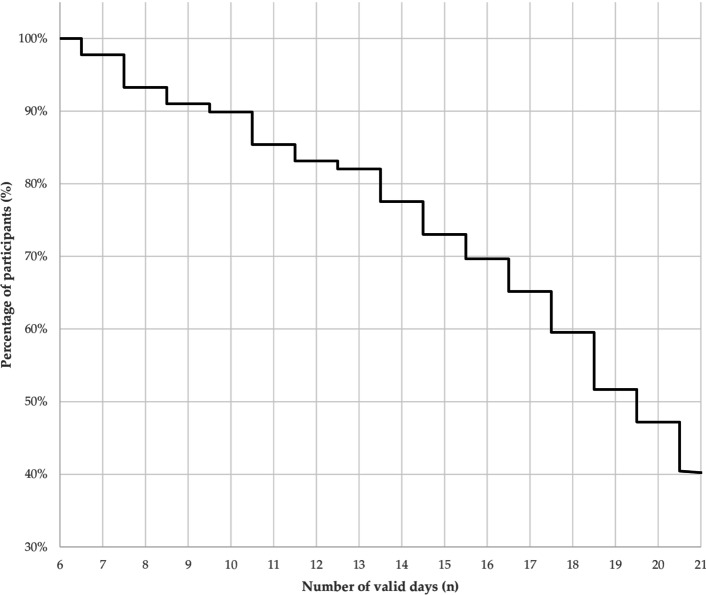
Kaplan Meier curve for measurement days per participants

On average, the measurement duration was 17.5 (± 5.1) days with ≥8 hours per day, see Table 1. Figure 3 shows the percentage of participants and the related measurement period. Hence, the majority (90%) of participants wore the smartwatch for at least 10 days.

Chi-squared test revealed no differences in the occurrence of the different days of the week (*p* = 0.59). Furthermore, 2nd degree polynomial regressions revealed no meaningful association between month of the year and neither of the component scores (gait: R^2^ <  0.05, *p* = 0.87; activity: R^2^ = 0.05, *p* = 0.23).

**Fig. 4:**
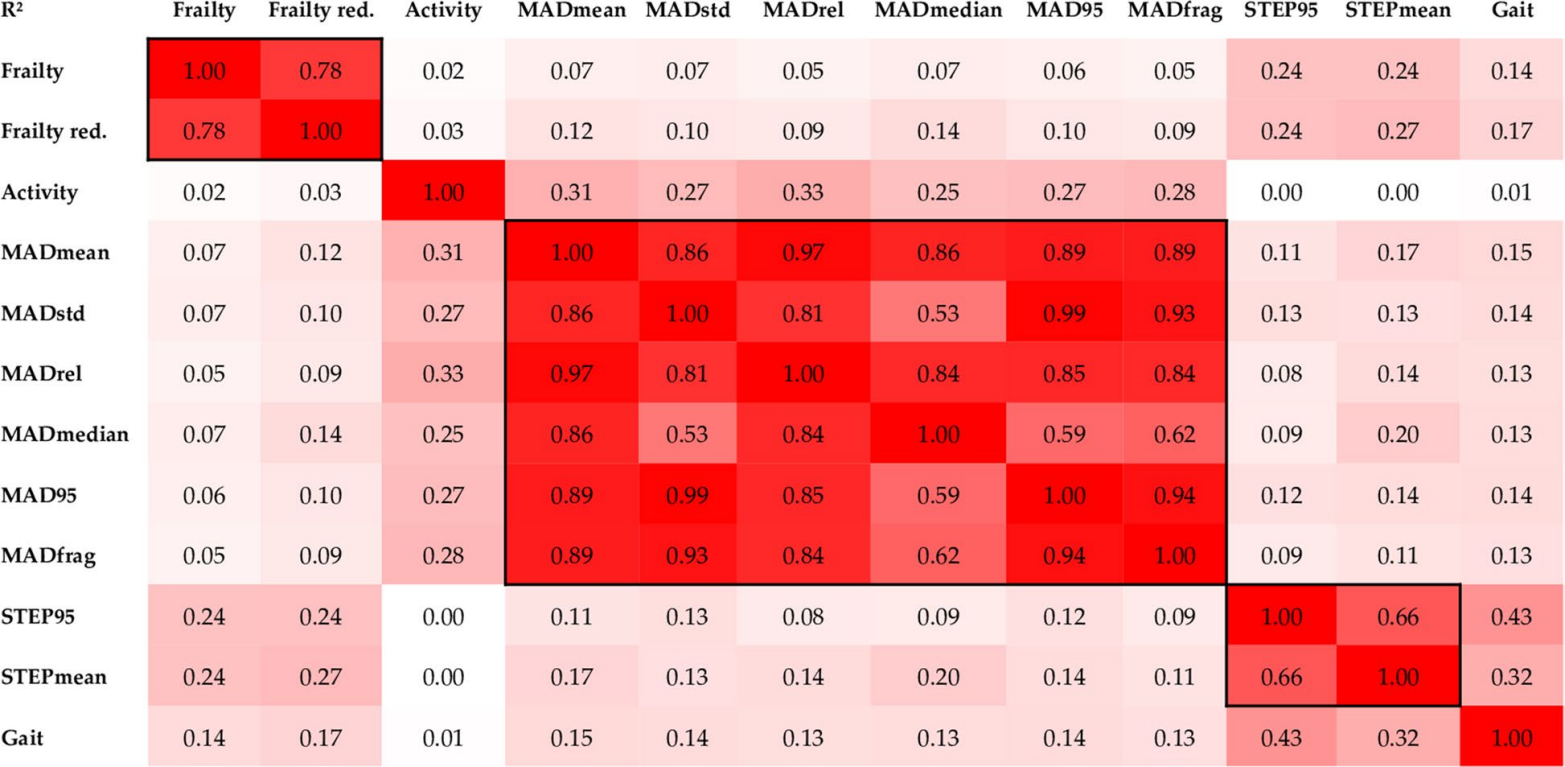
Correlation matrix (reporting the coefficient of determination R^2^) including all objective activity parameters, the subjective frailty scores, and the dimensions gait and activity derived from the confirmatory principal component analysis. *Frailty* 0:5, *Frailty red.* reduced frailty score 0:3, *MADmean* mean of all MAD values, *MADstd* standard deviation of all MAD values, *MADrel* relative amount time spend in MAD levels > 100 m-g, *MADmedian* median of all MAD values, *MAD95* 95th percentile of all MAD values, *MADfrag* standard deviation of the first derivate of the MAD time-series in milli-g, *STEP95* 95th percentile of cadence in steps per minute, *STEPmean* average number of steps taken per 5 s

**Fig. 5:**
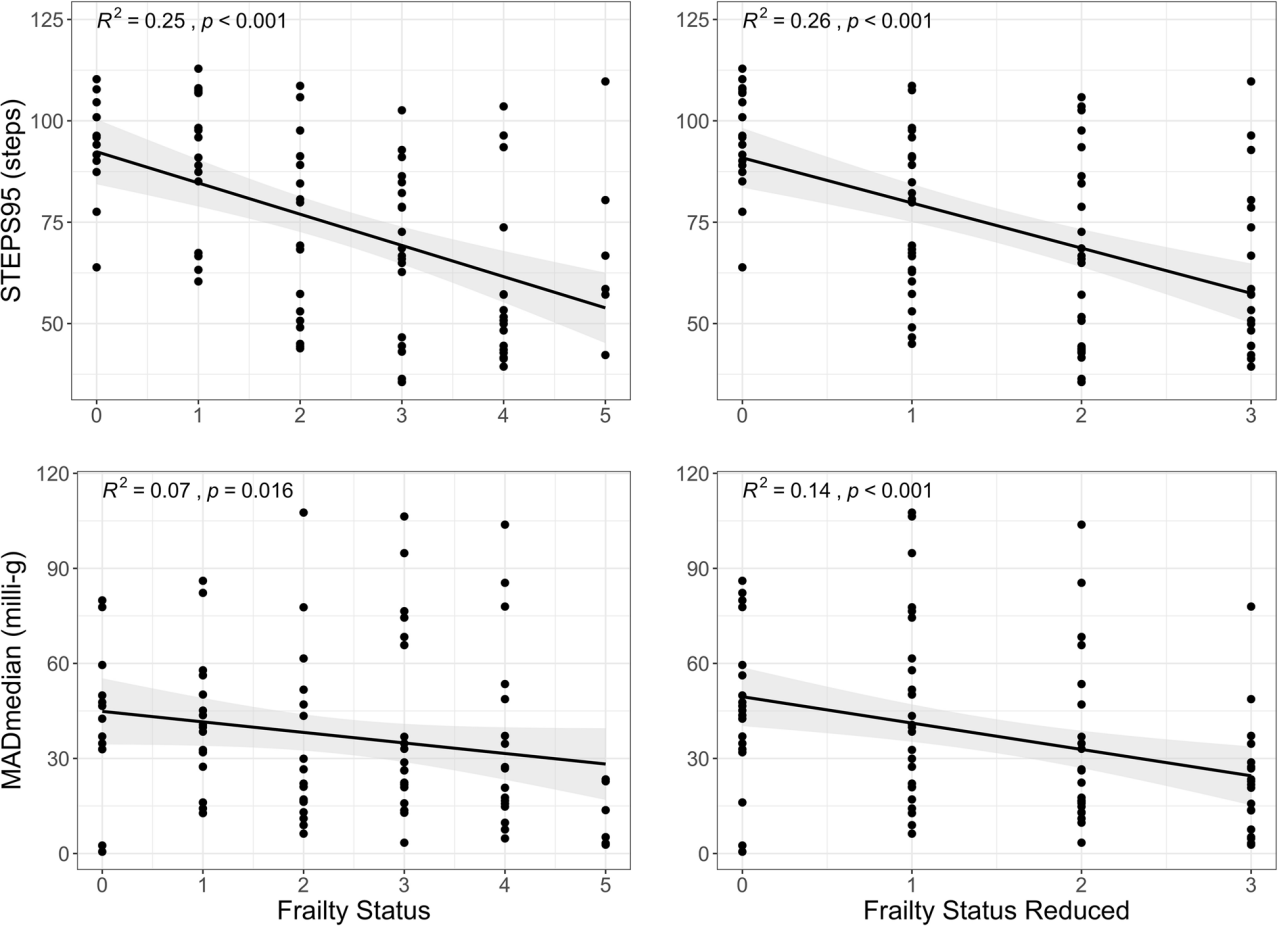
Both frailty scores (full 0:5, reduced 0:3) for the parameters STEP95 and MADmedian. *STEP95* 95th percentile of cadence, *MADmedian* median of all MAD values

Both, gait parameters and activity parameters, were highly correlated (Fig. 4). Therefore, only one parameter from each category was representatively correlated with the frailty scores (full score 0:5; reduced score 0:3). Correlation analyses revealed moderate negative correlations between the gait parameter ‘STEP95’ and both frailty scores (R^2^ = 0.25 & 0.26). Furthermore, weak to moderate negative correlations between the activity parameter (MADmedian) and both scores were found (R^2^ = 0.07 & 0.14). Figure 5 presents the scatterplots including the corresponding regression lines of the correlations. In addition, Fig. 4 shows the comparisons between the two surrogates (MADmedian & STEP95) and the other measures.

Table 2 illustrates the analyses of variance between objective activity parameters and the subjective frailty score criteria. The analyses showed significant differences with medium effects between the parameter ‘STEP95’ and the frailty criteria ‘weakness’ and ‘physical activity’ (both *p*-values ≤0.01, η^2^ = 0.10 - 0.12). This was also true for the dimension ‘gait’ (both p-values ≤0.01, η^2^ = 0.10 - 0.15). For MADmedian, the frailty criterion ‘physical activity’ revealed a significant difference with a medium effect (*p* ≤ 0.01, η^2^ = 0.10). The dimension ‘activity’ showed a significant difference for the frailty criterion ‘physical activity’ (p ≤ 0.01, η^2^ = 0.10).

**Table 2:** Comparisons between the objective parameters and the subjective frailty components

Objective parameters	Subjective frailty components	F value	p - value	Effect size, η^2^
STEP95 (steps)	Weight loss	3.277	0.074	0.04
	Muscle strength	5.926	0.017*	0.07
	Exhaustion	4.858	0.030*	0.06
	Weakness	8.983	0.004**	0.10
	Physical activity	11.402	0.001**	0.12
MADmedian (milli-g)	Weight loss	0.713	0.401	< 0.01
	Muscle strength	2.453	0.121	0.03
	Exhaustion	0.012	0.915	< 0.01
	Weakness	1.618	0.207	0.02
	Physical activity	9.238	0.003**	0.10
Activity	Weight loss	2.920	0.102	0.03
	Muscle strength	0.896	0.338	0.01
	Exhaustion	0.017	0.899	< 0.01
	Weakness	0.886	0.364	0.01
	Physical activity	9.136	0.003**	0.10
Gait	Weight loss	4.296	0.041*	0.04
	Muscle strength	4.253	0.042*	0.05
	Exhaustion	6.334	0.014*	0.07
	Weakness	14.627	< 0.001***	0.15
	Physical activity	8.728	0.004**	0.10

* ≤ 0.05, ** ≤ 0.01, *** ≤ 0.001. Effect size: eta-squared η^2^. *STEP95* 95th percentile of cadence, *MADmedian* median of all MAD values, *Unintentional weight loss* loss of appetite or decreased food intake, *Exhaustion* too little energy to execute daily tasks, *Low muscle strength* not able to carry objects of more than 5 kg, *Low physical activity* being engaged in at least moderate PA on less than once per week, *Weakness* not being able to take more than one flight of stairs without rest

The overall measure of sampling adequacy was 0.79. The Kaiser-Meyer-Olkin factor adequacy of the parameters was within the range of 0.61 - 0.92. The principal component analyses including the two components (activity and gait/ambulation) revealed 0.92 explained variance, and communalities of 0.99 for MADmean, 0.92 for MADstd, 0.96 for MADrel, 0.95 for MAD95, 0.79 for MADmedian, 0.94 for MADfrag, 0.91 for STEP95, and 0.91 for STEPmean. The subsequent cluster analysis of the participants’ component scores resulted in three clusters (number of clusters based on scree plot). The acceleration-based and person-related outcomes are reported in Table 3 including statistical comparisons of the three clusters. The statistical differences between the resulting clusters with regards to the frailty score and reduced frailty score are presented in Fig. 7. Regarding the application side of the watch (right or left wrist), 24 participants (69%) in cluster 1, 32 participants (97%) in cluster 2, and 3 participants (85%) in cluster 3, wore the watch on the left wrist (Chi-squared test: *p* = 0.008; V = 0.33). Post hoc comparisons (Fisher’s exact test) revealed a significant difference between cluster 1 and cluster 2 (*p* = 0.02). 

**Fig. 6:**
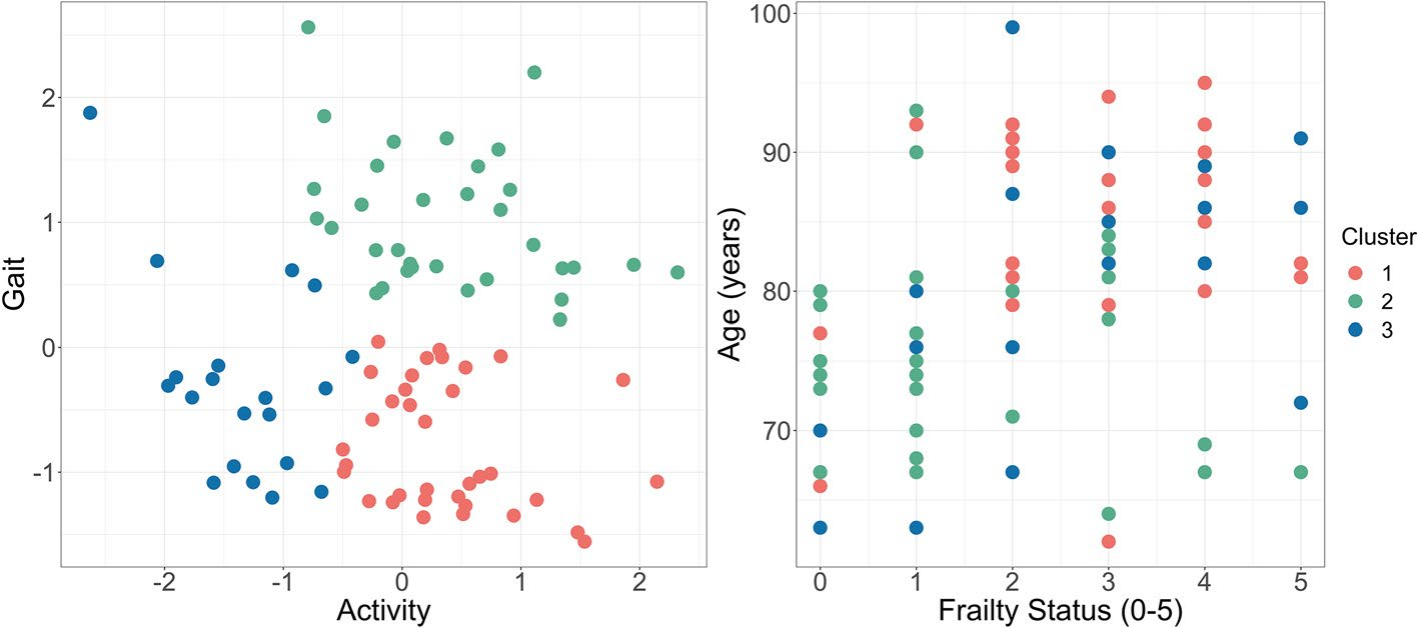
Individuals’ component scores in relation to ‘gait’ and ‘activity’ (left side) and in relation to age and self-reported frailty status (right)

Cluster 1 included 35 older adults, cluster 2 included 33, and cluster 3 had 20 persons included. By sorting and weighting (PCA) the activity data, two dimensions could be confirmed (cumulative variance of 0.92); activity and gait (ambulation). As a result, there were three different types of behavior - cluster: (1 - red) participants with high activity and low extent of ambulation, (2 - green) participants showing high activity and high extent of ambulation, and (3 - blue) participants with low activity and low extent of ambulation (see Fig. 6, left scatterplot). Analyses between the clusters showed statistically significant differences for the variables activity, gait, age, sex, number of chronic diseases, current state of health, and the use of a walking aid. Gait and activity showed high effect sizes between f^2^ 1.07 - 1.40. All clusters were comperable concerning their BMI, the comparison to the health status one year ago (course of health), and feeling of security inside and outside the house (Table 3). Figure 6 shows the three different clusters (indicated by different colors) in relation to acceleration-based activity and gait component scores (Fig. 6, left side) and in relation to age and self-reported frailty status (Fig. 6, right side).

**Table 3:** Acceleration-based and person-related outcomes stratified by cluster

Variables	Cluster 1	Cluster 2	Cluster 3	Comparison	Post hoc
	*n* = 35	*n* = 33	*n* = 20		*p* value, effect size
Activity	0.39 (0.64)	0.40 (0.80)	−1.34 (0.55)	*p* < 0.001, f^2^ 1.07	2-3: *p* < 0.001, d 2.43 1-3: *p* < 0.001, d 2.84
Gait	−0.79 (0.51)	1.02 (0.55)	−0.30 (0.76)	*p* < 0.001, f^2^ 1.40	1-2: *p* < 0.001, d − 3.42 2-3: *p* < 0.001, d 2.07 1-3: *p* = 0.012, d − 0.80
Age (years)	85.0 (8.0)	76.3 (7.5)	80.1 (10.1)	*p* < 0.001, f^2^ 0.47	1-2: *p* < 0.001, d 1.12
Female, n (%)*	25 (71)	15 (45)	8 (40)	*p* = 0.033 V 0.28	1-2 *p* = 0.03, OR 2.93 95% CI 1.08-8.35 1-3 *p* = 0.03, OR 3.63 95% CI (1.15-12.22)
BMI (kg/m^2^)	27.3 (5.5)	25.8 (3.2)	26.1 (4.5)	*p* = 0.374, f^2^ 0.15	–
Multimorbidity	1.6 (0.7)	1.0 (0.8)	1.5 (1.3)	*p* = 0.013, f^2^ 0.33	1-2: *p* = 0.014, d 0.80
Health Status**	3.6 (0.9)	3.0 (0.8)	3.8 (0.9)	*p* = 0.006, η^2^ 0.10	2-3: *p* = 0.022, *d* − 0.90 1-2: *p* = 0.019, *d* − 0.71
Course health status**	3.5 (0.9)	3.2 (1.0)	3.6 (0.9)	*p* = 0.294, η^2^ 0.01	–
Feeling of safety (home)**	1.5 (0.7)	1.6 (0.7)	1.7 (1.0)	*p* = 0.637, η^2^ ‘ -0.01	–
Feeling of safety (outside)**	2.5 (1.2)	2.0 (0.8)	2.8 (1.6)	*p* = 0.145, η^2^ 0.02	–
Walking aid, n (%)*	28 (80)	4 (12)	13 (65)	*p* < 0.001, V 0.62	2-3: *p* < 0.001, OR 12.34 95% CI 3.27-57.69 1-2: *p* < 0.001, OR 26.25 95% CI 7.55-116.66
Frailty Status, n (%)*				*p* = 0.21, V 0.18	–
Robust	3 (9)	7 (21)	2 (10)		–
Pre-frail	11 (31)	15 (45)	7 (35)		–
Frail	21 (60)	11 (33)	11 (55)		–

Mean values, standard deviations. Effect sizes: Cramer’s V, Cohen’s d, Cohen’s f^2^ eta-squared η^2^, Cliff’s Delta *d*, * tested by Chi test and OR comparison, ** tested by Kruskal-Wallis test. *BMI* body mass index, *Multimorbidity* number of comorbidities (0 - 9), *Health status* self-rated (best: 1; worst: 5), *Course health status* self-rated comparison to health 1 year ago (improvement: 1; deterioration: 5), *Feeling of security* 1: fully; 5 not at all

When comparing activity and gait between the clusters, cluster 2 (green) appeared to contain the most active group in both categories; activity as well as gait (ambulation). The distribution in relation to age and frailty status shows a location primarily in younger age and lower levels of subjectivly reported frailty status (see Fig. 6, right). This is supported by statistical comparison showing age differences between cluster 1 and 2 (Table 3). Cluster 3 (blue) represented the most inactive group indicated by low extent of ambulation and low activity. In relation to age and frailty status, cluster 3 showed a more intermediate distribution across all ages and frailty levels (see Fig. 6, right side) with no significant difference in comparison to the other clusters (cluster 1 and cluster 2). In comparison, cluster 1 was primarily represented by older paricipants with higher frailty status, but low ambulation and high activity. A post hoc analysis showed significant differences in the extent of ambulation (gait) between all 3 clusters (effect sizes from d − 3.42 - 2.07), with cluster 1 being the group with the lowest extent of ambulation followed by cluster 3 and cluster 2. Whereas for activity, differences appeared to be present only between cluster 1 and 3 and cluster 2 and 3. Cluster 1 and 2 showed comperable levels of activity. In general, the post hoc analysis confirmed the distribution into the different clusters. Additionally, the number of chronic diseases and self-reported health status differed between cluster 1 and 2. Self-reported health status differed between cluster 2 and cluster 3, see Table 3. The odds of being female in cluster 1 was significanlty increased with an OR of 3.63 (95% CI 1.15-12.22, *p* = 0.03). Consequently, the odds of being male in and allocated in cluster 2 or 3 was significanlty increased but did not differ from each other (*p* = 0.71). The odds ratio of being frail was increased for participants in cluster 1 (OR 2.93, 95% CI 1.10 - 8.22, *p* = 0.03) and cluster 3 (OR 2.39, 95% CI 0.76 - 7.82, *p* = 0.14), while only cluster 1 reached the level of significance. Overall, 38% of men used a walking aid in daily life, compared with 62% of women. Therefore, the odds of having a walking aid as women compared to men was significanlty increased with an OR of 2.73 (95% CI 1.16 - 6.68; *p* = 0.02). All smartwatch derived parameters are listed in Table 4.

**Table 4:** Smartwatch derived parameters across the three clusters

Parameters	Cluster 1 n = 35	Cluster 2 n = 33	Cluster 3 n = 20	Comparison	Post hoc p - value, effect size
MADmean (milli-g)	74.3 ± 18.8	86.3 ± 22.6	32.7 ± 12.3	*p* < 0.001, f^2^ 1.09	1 - 3: < 0.001, d 2.48 2 - 3: < 0.001, d 2.76 1 - 2: 0.03, d -0.58
MADstd (milli-g)	89.3 ± 12.2	96.2 ± 13.7	52.8 ± 12.1	*p* < 0.001, f^2^ 1.35	1 - 3: < 0.001, d 3.00 2 - 3: < 0.001, d 3.31 1 - 2: 0.08, d -0.53
MADrel (milli-g)	30.1 ± 8.0	33.6 ± 9.0	11.9 ± 5.6	*p* < 0.001, f^2^ 1.10	1 - 3: < 0.001, d 2.52 2 - 3: < 0.001, d 2.75 1 - 2: 0.20, d -0.41
MADmedian (milli-g)	36.8 ± 22.9	52.8 ± 25.5	11.4 ± 8.6	*p* < 0.001, f^2^ 0.73	1 - 3: < 0.001, d 1.33 2 - 3: < 0.001, d 1.98 1 - 2: < 0.01, d -0.66
MAD95 (milli-g)	252.4 ± 39.2	276.3 ± 44.9	131.9 ± 42.2	*p* < 0.001, f^2^ 1.36	1 - 3: < 0.001, d 2.99 2 - 3: < 0.001, d 3.29 1 - 2: 0.06, d -0.57
MADfrag (milli-g)	84.5 ± 12.2	89.8 ± 15.4	50.3 ± 12.9	*p* < 0.001, f^2^ 1.16	1 - 3: < 0.001, d 2.75 2 - 3: < 0.001, d 2.72 1 - 2: 0.25, d -0.38
STEP95 (steps)	58.3 ± 14.7	97.4 ± 9.0	63.5 ± 19.9	*p* < 0.001, f^2^ 1.28	1 - 3: 0.41, d -0.31 2 - 3: < 0.001, d 2.41 1 - 2: < 0.001, d -3.19
STEPmean (steps)	0.2 ± 0.1	0.9 ± 0.3	0.2 ± 0.2	*p* < 0.001, f^2^ 1.36	1 - 3: 0.93, d 0.00 2 - 3: < 0.001, d 2.62 1 - 2: < 0.001, d -3.17

Mean values, standard deviations. Effect sizes: Cohen’s d, Cohen’s f^2^ *MADmean* mean of all MAD values, *MADstd* standard deviation of all MAD values, *MADrel* relative amount time spend in MAD levels > 100 m-g, *MADmedian* median of all MAD values, *MAD95* 95th percentile of all MAD values, *MADfrag* standard deviation of the first derivate of the MAD time-series in milli-g, *STEP95* 95th percentile of cadence in steps per minute, *STEPmean* average number of steps taken per 5 s

The analysis of separate odds ratios for the reported frequency of frailty components, showed that especially cluster 1 (primarily older women) had an increased risk for low PA (OR 6.67, 95% CI 1.86 - 20.28, *p* < 0.01) and for weakness (OR 3.65, 95% CI 1.27 - 11.43, *p* < 0.01). Additionally, there was a trend towards increased exhaustion for this cluster (OR 2.33, 95% CI 0.88 - 6.46, *p* = 0.09), see Table 5. The most frail group (cluster 3) had an increased risk for low PA (OR 6.46, 95% CI 1.82 - 26.29, *p* < 0.01) and showed tendencies towards increased weakness (OR 2.73, 95% CI 0.83 - 10.29, *p* = 0.09) as well as for low muscle strength (OR 2.72, 95% CI 0.85 - 9.54, *p* = 0.08).

**Table 5:** The odds ratios for each self-reported frailty criteria across the three clusters

Frailty Criteria	Frequency, n (%)	OR	95% CI	p - value
Unintentional weight loss				
Cluster 1	13 (37)	1.56	0.56 - 4.53	0.38
Cluster 3	6 (30)	1.15	0.32 - 3.96	0.83
Exhaustion				
Cluster 1	19 (54)	2.33	0.88 - 6.46	0.09
Cluster 3	8 (40)	1.33	0.41 - 4.29	0.64
Low muscle strength				
Cluster 1	19 (35)	1.42	0.54 - 3.76	0.47
Cluster 3	14 (54)	2.72	0.85 - 9.54	0.08
Low physical activity				
Cluster 1	18 (51)	6.67	1.86 - 20.28	< 0.01**
Cluster 3	11 (55)	6.46	1.82 - 26.29	< 0.01**
Weakness				
Cluster 1	28 (80)	3.65	1.27 - 11.43	0.01*
Cluster 3	15 (75)	2.73	0.83 - 10.29	0.09

Cluster 2 was used as reference (Chi^2^ test). *OR* Odds ratio, *Frequency* frequency of frailty components, * < 0.05, ** < 0.01, *** < 0.001. *Unintentional weight loss* loss of appetite or decreased food intake, *Exhaustion* too little energy to execute daily tasks, *Low muscle strength* not able to carry objects of more than 5 kg, *Low physical activity* being engaged in at least moderate PA on less than once per week, *Weakness* not being able to take more than one flight of stairs without rest

**Fig. 7:**
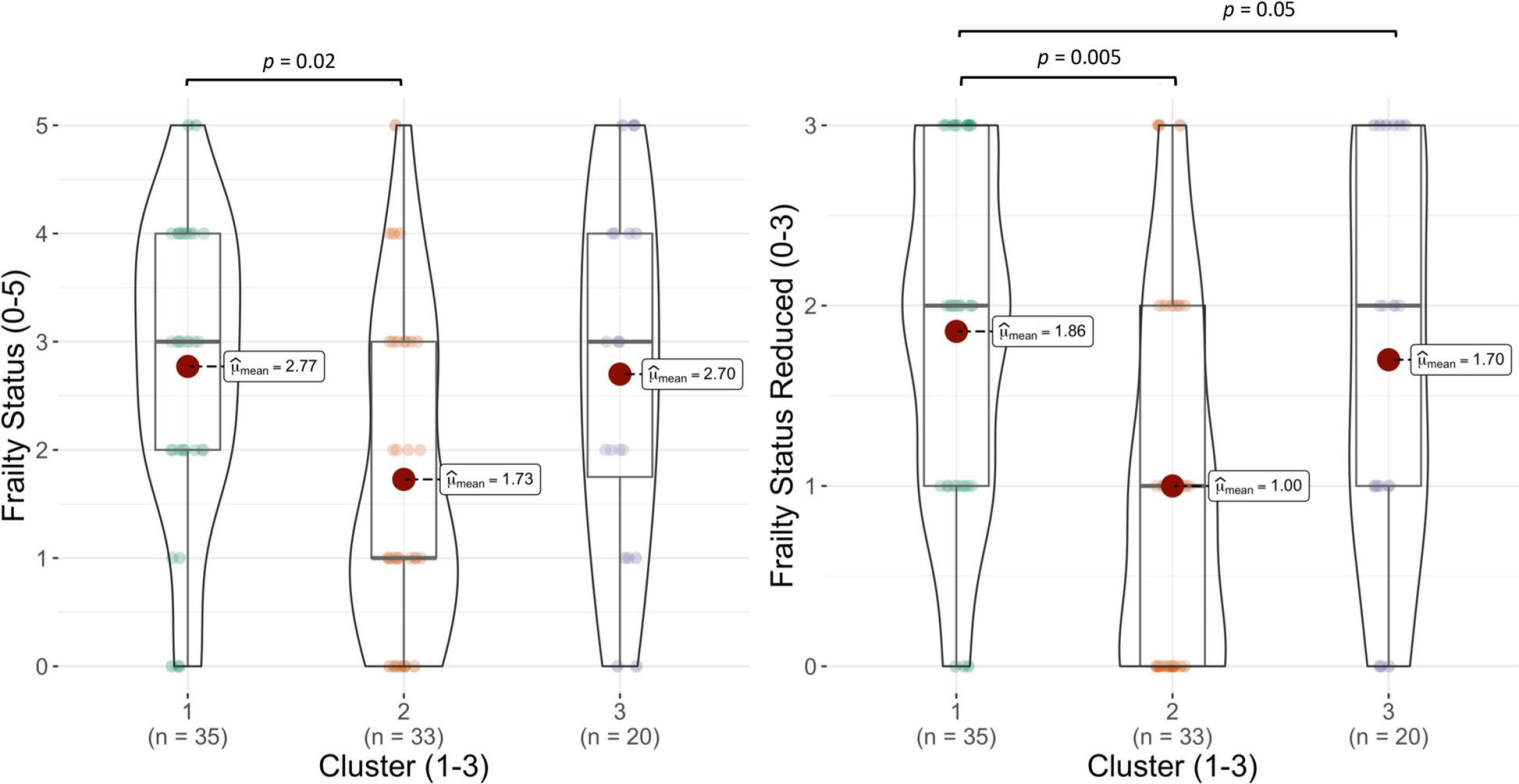
Mean scores of both self-reported frailty scores (full 0:5, reduced 0:3) between the three clusters

Analysis of variance revealed a significant difference with a large effect between the full frailty status (0:5) and the clusters (*p* = 0.007, f^2^ = 0.35), see Fig. 7. Post hoc analyses showed a significantly higher level of frailty in cluster 1 as compared to cluster 2 (1 - 2: *p* = 0.02). The comparison of cluster 1 and cluster 3 did not reach significance, but showed a trend towards higher frailty scores in cluster 1 (1 - 3: *p* = 0.06). When running the same analysis with the reduced version of the frailty score (0:3), again, significant differences with a large effect between the clusters were found (*p* = 0.002, f^2^ = 0.40), see Fig. 7. Post hoc analyses revealed that participants in cluster 1, on average, had significantly higher frailty scores when compared to participants allocated to cluster 2 (1 - 2: *p* = 0.005). And again, we observed a tendency towards higher frailty scores in cluster 1 compared to cluster 3 (1 - 3: *p* = 0.05).

## Discussion

In this study, we examined smartwatch-derived characteristics of everyday behavior (physical activity) and subjective frailty status in older adults. The main objective was to investigate and better understand the relation between sensor-based measures of daily physical activity, separated into upper limb activity and gait (ambulation), and the self-estimation of frailty levels. The correlation of the derived components gait and activity with the self-reported frailty scores were weak to moderate. Cluster analysis resulted in two clusters with either low (cluster 3) or high activity (cluster 2) in the dimensions gait and activity. The third cluster (cluster 1) was characterized by high activity and low extent of gait. The odds of being female and frail was significantly increased for cluster 1. Additionally, the odds of having a walking aid as women compared to men was increased, too. 

The correlation analyses between the frailty level of our cohort (exclusively self-reported), showed moderate negative associations with the gait parameter ‘STEP95’ (cadence-based). Additionally, weak to moderate negative correlations were found between the activity parameter ‘MADmedian’ (MAD-based) and both scores. The difference between the two frailty scores was that we excluded the criteria ‘muscle strength’ and ‘weight loss’ for the reduced version (0:3) to examine whether the explained variance increased when we omitted these parameters, which may not be directly measurable with a wrist-worn sensor. In general, however, both scores revealed comparable results. A more direct comparison between the objective physical activity data and the subjective frailty components was done by separate analyses of variance. Both, the parameter ‘STEP95’ and the dimension ‘gait’ showed differences with medium effects for the criteria ‘weakness’ and ‘physical activity’. The upper limb related activity (‘MADmedian’ and ‘activity’) showed differences with a medium effect for the ‘physical activity’ item. These results seem to be consistent with the content of the questions. The ‘physical activity’ question includes all types of physical activity, regardless of whether the upper or lower limbs are affected. However, the ‘weakness’ question specifically asks about the ability to climb one or more flights of stairs. Furthermore, the dimension ‘gait’ showed additional small effects for the criterion ‘weight loss’ and ‘exhaustion’. Our findings are consistent with previous studies stating that the level of activity (respectively inactivity) is associated with different frailty levels [18–21, 23, 24] and that especially gait (ambulation) related parameters seem to be more sensitive [18, 19]. In 2012, Theou and colleagues [18] presented a wide range of comparisons between different PA measures (e.g., accelerometer, heart rate (HR), electromyography (EMG), global positioning system (GPS), and Minnesota Leisure Time Physical Activity Questionnaire (MLTAPQ)) with each other and with the Frailty Index (FI). They found that the FI was significantly correlated to all PA measures. For accelerometry, ‘total steps’ and ‘total activity minutes’ were most strongly correlated to FI [18]. Our study extends this body of evidence by showing that there is also a correlation, however only weak to moderate, between an exclusively self-reported frailty score, including five short and easy questions, and gait and activity-related parameters as assessed by accelerometry. Furthermore, for our parameters related to gait, a significant difference was found for almost all frailty criteria, suggesting that mobility might be the driving parameter related to frailty, although the relationship between frailty and behavior might be multimodal, as seen, for instance, in the relation with falls [36]. Still, about 60% of the variance of behavior and the FI frailty assessment [18] (mixed assessment) as well as approximately 75% of the comparison with the self-reported frailty questionnaire remains unexplained. In addition, it should be considered that the association between pedometry and self-reports may be due to the fact that the self-report explicitly asks for gait function. However, R^2^ did not change when non-PA questions were removed, indicating a generalizability of ambulation (via pedometry) to other domains. 

The debate about the relationship between objective and subjective activity assessments in older adults is well known (e.g., [13, 37, 38]). The lack of complete agreement could indicate insufficient validity of self-reports or objective assessments, or (alternatively) low sensitivity of self-reports and objective measures [39]. This may include various aspects, such as detail of the questionnaire, extent of supervision, or the length of the recall period. Questionnaires may cover periods from one to seven days, or even up to several months. Answers are dependent on the subjects’ age, their living environment as their health/behavioral reference (e.g., when an old person compared him/herself with the younger neighbor), and the context of questioning [40]. In contrast to self-reports, objective assessments using wearable sensors can provide accurate documentation of daily activities such as walking, standing, sitting, and lying down [24, 25], which in turn, may allow to identify frailty-specific patterns in peoples’ natural environment [23]. Different levels of frailty may be characterized by differences in daily PA patterns, such as fragmented walking distances (e.g., due to exhaustion, declining strength, or walking indoors instead of outdoors) or lower PA complexity [23]. However, in addition to the simple comparison of objective and subjective measures, cluster analyses might contribute to the validation of self-reports as a new approach and, in this sense, this may lead to a better understanding of what the self-report measures assess. 

Based on the behavioral data (acceleration data), our cluster analysis resulted in three clusters that differed in terms of their upper extremity pronounced activity level and extent of gait (ambulation). Cluster 2 appeared to represent the most active group showing the highest extent of ambulation and activity, followed by cluster 1 (high activity and low extent of ambulation) and the least active cluster 3 (low activity and low extent of ambulation). Participants allocated to cluster 2 did not only show a different extent of gait and activity level, but also had a better self-rated health status (mean = 3.0; i.e., ‘good’) and less frequent use of walking aids. In contrast, participants in cluster 1 and cluster 3 showed a comparable use of walking aids. Therefore, the odds ratio of using walking assistive devices was significantly increased for cluster 1 and 3 compared to cluster 2. Yet, 33% of the participants classified into cluster 2 were categorized as frail and 45% as pre-frail. Our analyses of variance further revealed significant differences between the clusters and the frailty scores (both full and reduced version). Post hoc analyses showed that the clusters in which participants showed lower extent of ambulation also contained subjects with a higher frailty score (cluster 1 and cluster 3). This led to one robust group (cluster 2) and two frailty subtypes. However, for elderlies allocated to cluster 1, not only the risk of being frail was significantly increased, but also the probability of being female. Only the parameter ‘gait’ showed clear differences between all three clusters with a large effect. Consequently, cluster 1 contained the participants with the highest frailty scores, most of whom were female, and on average the oldest group. Interestingly, this finding seems to be consistent with the ‘male-female health-survival paradox’, which states that women live longer than men, but with poorer health [41] as they usually experience more functional limitations, co-morbidities, and poorer self-rated health [42, 43]. Studies using the FI consistently show that women have higher FI scores than men at all ages even though they tolerate a higher degree of frailty (lower mortality rate at any given FI score or age). Therefore, they can be seen as more frail (due to a poorer health status) and less frail (lower risk of mortality) at the same time [41]. Gender differences also seem to be reflected in activity behavior. In a study of Li et al. [44] PA was assessed by the CHAMPS (Physical Activity Questionnaire for Older Adults) and accelerometry for 7 consecutive days. They analyzed the data of 114 older adults (mean age 74.0 ± 6.0) and observed that preferences for level, type, and location of PA differed substantially between gender [44]. In addition, there is evidence based on sensor measures that men engage in more MVPA than women (e.g., [45, 46]), and that there might be also gender differences in the amount of time spent in lower intensity domains, such as sedentary and light activities [47]. Accordingly, even though men might achieve a greater amount of MVPA, they spend more time sedentary, whereas women may accumulate a greater number of light activities [17, 46, 47]. This fits quite well to the results of our cluster analysis showing that cluster 1 (most frail participants and 71% female) still performed activities connected to upper limb movements. Light housework (e.g., dusting, sweeping), or even cooking, body hygiene, and knitting, for example, may therefore have led to increased hand activity in women in our cohort. Additionally, potential gender issues may be present in the use of walking aids as well. There is evidence showing that predominantly women use walking aids, e.g., [48]. Consequently, this may have had an effect in the subjective frailty scoring, too. The frail group, based on the subjective frailty score, included 67% women and 72% walking aid users. However, for the results of the cluster analysis, there was no significant difference between cluster 1 and 3 regarding the use of walking aids. 

Considering cluster 2 as the robust group – in terms of gait (ambulation) and upper limb PA, the differences between the two frail groups (1 and 3) remain to be discussed. Cluster 3 showed what the stereotype of frailty suggests: inactivity in both the gait (ambulation) and the upper limb PA dimension. The odds ratio analysis of each separate criterion revealed that especially participants within cluster 3 reported low PA and showed a trend towards increased weakness i.e., ‘climbing stairs’ and tendency towards low muscle strength i.e., ‘lifting a heavy bag’. Cluster 1 was prone to both a high risk of low PA and weakness. Moreover, participants in cluster 1 showed a tendency towards exhaustion i.e., ‘lack of energy’. Additionally, the parameter MADfrag, which reflects the relation of long and short activity bouts, and therefore displays the specific changes in intensities, was particularly low for people allocated to cluster 3. This is in line with our previous study in which we found that higher frailty scores were associated with more monotonous behavior during two activities of daily living (gardening and tea task) [35]. To what extent the use of walking aids might be seen as a consequence of frailty or might lead to the classification as frail, could be answered by cluster 1. Participants in this cluster walked even less than the other frail group (cluster 3), while showing the same upper limb related activity rate as the robust group. This group (cluster 1) had walking aids in 80% of cases. Thus, assuming that pedometers have problems detecting steps in people using walkers [26], analyzing walking activity alone might not provide valid information about frailty. Additionally, the questions related to ‘weakness’ in the questionnaire used in our study (equivalent to ‘walking speed’ of the Fried frailty score [9]) was about difficulties going up one or several flights of stairs without resting. This tends to basically exclude people with walking aids as it becomes more difficult to climb stairs and consequently might result in a positive rating for this criterion. This is in line with existing evidence. Nunes et al. [15], for example, showed in their study that comparing the Fried frailty criteria with a self-reported frailty questionnaire, ‘decreased walking speed’ showed a rather low specificity of 31.4%. Therefore, gait-related self-reported information may be misleading when it comes to frailty. Barreto and colleagues [25] investigated a self-reported frailty screening tool at baseline and one year later. Their analysis showed that frail individuals were older, predominantly female, had more co-morbidities, a greater decline in physical function, suffered more often from chronic pain, and reported decreased health status. Furthermore, they stated that frailty is a single entity, different from co-morbidity and physical limitation [25]. The question that arises from the train of thoughts is whether a person remains active, regardless of physical or neurological impairments that might impair walking. Although the values of the metabolic equivalent of task (MET) differs between gait and upper limb related activities, motor function of upper extremity has been identified as an important predictor of, e.g., disability [49]. In general (in terms of METs), lower body activities should have higher energy expenditure in comparison to upper body activities because they involve major muscle groups and the whole body mass is moving (instead of just an arm). Therefore, it could be meaningful to investigate changes in the different categories (ambulation and upper body activity), as changes in physical behavior may represent the first sign of frailty. This leads to the idea of a parameter for early detection of frailty. If we assume that cluster 1 is self-classified as frail solely due to ambulatory impediments, more specific actigraphy assessments of everyday behavior could help to get more information on the overall activity level of the person as well as to differentiate between frailty and possible frailty-biases (e.g., using walking assistive devices or questions, which disadvantage people with walking aids). This could ultimately help to identify causes and mechanisms of what we consider physical frailty. However, in our study, the number of robust older adults was far less than the pre-frail and frail older adults. This might be due to the fact, that the frailty classification was solely based on the self-estimation of the individuals. Consequently, the participant may have considered themselves in a worse condition than they are. 

This study has several strengths, but also limitations that need to be addressed and therefore, results should be interpreted with caution. First, we intended to increase the compliance and wear time of the smartwatch by using the wrist of choice of the participants [24, 26]. This further allows for continuous data collection and the capturing of more commonly performed tasks of daily living [26], including gait activity. This resulted in significant group differences between placement on the left or right wrist between cluster 1 and cluster 2. However, this may not have been of relevance, as these clusters differed in the extent of ambulation (gait), but not in the amount of hand-related activity. It is possible that this is a consequence of wearing the watch in a balanced proportion on the non-dominant or dominant hand between both groups, or more bimanual hand activity [50]. While this is solely based on speculation, as we had no information about hand dominance, activity in cluster 1 might have been overestimated due to more frequent measurement on the dominant hand compared to cluster 2. With regard to future studies, we recommend to assess handedness and define where the sensor should be worn. Second, while most clinical tests are geared towards measuring maximum physical performance (capacity), the use of wearables in everyday life, in contrast, is aimed at recording actual (submaximal) behavior. However, individuals’ submaximal behavior and its relation to capacity could deviate. This is usually the case unless the capacity is greatly reduced and thus no longer different from actual everyday behavior. Therefore, the interpretation of the actual behavior remains difficult. In addition, accelerometry (in the proposed form) is not able to detect the movement of non-body masses (e.g., carrying groceries) and could therefore underestimate the energy expenditure. Third, devices worn on the wrist are known to be susceptible to interference with the use of assistive devices such as walkers [26, 51], which is increasingly the case in older age. In this context, especially the use of rolling walkers leads to a rather stationary wrist, and it is still unclear how much movement is actually registered by the device [26]. Although we assessed whether participants used an assistive device, we did not further subdivide the assistive devices (e.g., walking sticks or rolling walkers). For future studies, the simultaneous use of hip or ankle pedometers and wrist-worn bands might be an attractive solution, since wearables are becoming smaller and cheaper. If such instruments were combined, this could also provide more insights into the importance of energy consumption during certain activities. Another limitation refers to the smartwatch itself. As we had some major issues with the charging process and data collection, some participants were not able to handle the smartwatch independently and dropped out of the study. Consequently, the dropout rate was around 40%, which is quite high. This also affected the wear time of the watch drastically and we had to adapt the valid measurement hours to decrease data loss and the burden for the participants. The current wear time recommendation for valid accelerometer measurements is ≥10 hours/day for 6-9 days (e.g., [28]). Since we had issues with the frequent charging process of the watch, we adapted the amount of valid measurement hours down to ≥8 hours to decrease the burden for the participants. However, since we had an extended wearing period of at least 10 days for 90% of the sample, this might still achieve good reliability in capturing PA. Another limitation which needs to be discussed is the use of a convenience sample and therefore the lack of generalizability. Due to the potential bias of the sampling technique, subgroups might be under-represented, e.g., those who were not interested in the topic (public dissemination). In this regard, care managers were involved in the recruitment process to reach out for people in nursing homes as well. For future research, we would strongly recommend the use of devices with higher battery life and greater robustness towards unintentional adjustment of measurement setting.

## Conclusion

This multicentric cross-sectional study showed that simple correlation analyses of smartwatch derived parameters (based on MAD and steps) and a self-reported frailty score leave up to 75% variances unexplained. However, cluster analysis showed a meaningful differentiation between three clusters based on the extent of gait (ambulation) and upper limb PA during everyday life. Interestingly, especially one group (cluster 1) had a higher risk of self-reported frailty, being older, female, and dependent on walking aids, despite showing hand activity. To summarize, while subjective frailty assessments may be a simple first screening approach, older women using walking aids appear to classify themselves as frail more frequently, despite still being physically active. Therefore, self-reports may be particularly biased in older women and, thus, actigraphy or the use of additional sensors may be necessary to get comprehensive information about their physical status.

## Abbreviations

MAD: Mean amplitude deviation
MADmean: Mean MAD
MADstd: Standard deviation of MAD
MADrel: Relative MAD
MADmedian: Median MAD
MAD95: 95th percentile of MAD
MADfrag: Fragmentation rate of MAD
STEP95: 95th percentile of cadence
STEPmean: Average number of steps per 5 s
PA: Physical activity
HR: Heart rate
EMG: Electromyography
GPS: Global positioning system
R: Robust
P: Pre-frail
F: Frail
OR: Odds ratio
CI: Confidence interval
BMI: Body mass index
MLTPAQ: Minnesota Leisure Time Physical Activity Questionnaire
MVPA: Moderate to vigorous physical activity
FI: Frailty Index
CHAMPS: Physical Activity Questionnaire for Older Adults
MET: metabolic equivalent of task
